# Risk factors for sudden cardiac arrest in patients with ST-segment elevation myocardial infarction: a retrospective cohort study

**DOI:** 10.1186/s12873-022-00732-3

**Published:** 2022-10-24

**Authors:** Chang-Hung Chu, Hong-Mo Shih, Shao-Hua Yu, Shih-Sheng Chang, Ji-Syuan Sie, Fen-Wei Huang, Tai-Yi Hsu

**Affiliations:** 1 School of Medicine, College of Medicine, China Medical University, No. 91, Xueshi Rd., Taichung, 404 Taiwan; 2 Department of Emergency Medicine, China Medical University Hospital, No. 2, Yude Rd., Taichung, 404 Taiwan; 3 Department of Public Health, China Medical University, No.100, Section 1, Economic and Trade Rd., Taichung, 406 Taiwan; 4 Graduate Institute of Biomedical Sciences, China Medical University, No. 91, Xueshi Rd., Taichung, 404 Taiwan; 5 Division of Cardiovascular Medicine, China Medical University Hospital, No. 2, Yude Rd., Taichung, 404 Taiwan

**Keywords:** Sudden cardiac arrest, ST-segment elevation myocardial infarction, Percutaneous coronary intervention, Coronary artery disease

## Abstract

**Background:**

Sudden cardiac arrest (SCA) is a critical complication of acute myocardial infarction, especially ST-segment elevation myocardial infarction (STEMI). This study identified the risk factors for SCA in patients with STEMI before receiving catheterization.

**Methods:**

We retrospectively analyzed the data of patients with STEMI and cardiac arrest who presented to a tertiary care center in Taiwan between January 1, 2016, and December 31, 2019. Only patients with coronary artery disease (CAD) confirmed by coronary angiography were included in this study. We collected the patients’ demographic and clinical data, such as age, sex, medical history, estimated glomerular filtration rate (eGFR), and coronary angiographic findings. The primary outcome of this study was SCA in patients with STEMI. Continuous and nominal variables were compared using the two-sample Student's *t*-test and chi-squared test, respectively. The results of logistic regression were subjected to multivariate analysis with adjustment for possible confounders.

**Results:**

A total of 920 patients with STEMI and coronary angiography–documented CAD and 108 patients with SCA who presented between January 1, 2016, and December 31, 2019, were included. The bivariate logistic regression analysis of patients’ demographic data revealed that patients with STEMI and SCA were slightly younger, were more likely to have diabetes mellitus, and had a lower eGFR than did the patients without SCA. The coronary angiographic findings indicated a higher prevalence of left main CAD and three-vessel disease in patients with SCA than in patients without SCA. Multivariate logistic regression revealed that left main CAD (odds ratio [OR]: 3.77; 95% confidence interval [CI], 1.84 to 7.72), a lower eGFR (OR: 0.97; 95% CI, 0.96 to 0.98), and younger age (OR: 0.98; 95% CI, 0.96 to 0.99) were the risk factors for SCA in patients with STEMI.

**Conclusions:**

Left main CAD, lower eGFR, and younger age are the risk factors for cardiac arrest in patients with acute myocardial infarction.

## Background

Sudden cardiac arrest (SCA) contributes to 50% of cardiovascular deaths worldwide, with a recently estimated incidence of 4 to 5 million cases annually [1]. Coronary heart disease (CHD) is the most common pathology underlying SCA [2]. In patients with CHD, no warning signs occur before an SCA episode, which is often caused by asystole or ventricular fibrillation [3]. CHD is responsible for 7% of deaths among men worldwide. In approximately 5% of CHD cases, a myocardial infarction causes cardiac arrest [4]. CHD is characterized by acute myocardial infarction (AMI), the main reason for SCA occurrence and disability worldwide [5]. AMI is divided into three subgroups, namely ST-segment elevation myocardial infarction (STEMI), non–ST-segment elevation myocardial infarction (NSTEMI), and unstable angina.

Zimmermann et al. reported that the predictors of poor outcomes in patients with out-of-hospital cardiac arrest (OHCA) and STEMI include increased age, reduced ejection fraction at hospital admission, initial asystole rhythm, renal insufficiency, delayed cardiopulmonary resuscitation (CPR), and delayed return of spontaneous circulation (ROSC) [6]. The risk of SCA is high in patients with coronary artery disease (CAD), structural heart disease, clinical heart failure, prior aborted cardiac arrest, and left ventricular ejection fraction less than 30% [2]. Bertic et al. reported that patients with STEMI and OHCA had higher in-hospital cardiac arrest rates, cardiogenic shock incidence, and mortality than those without OHCA in Vancouver Coastal Health Authority Hospital [7]. In India, Rao et al. reported that older age, female sex, severe left ventricular dysfunction, noncompliance with medication, and the absence of both reperfusion and revascularization are the predictors of a high risk of sudden cardiac death [8]. No clinical study has focused on the association between STEMI and SCA in Taiwan. A previous study only reported patterns of AMI in Taiwan from 2015 to 2019 [9].

Because of the disappointing survival outcome in patients with STEMI and SCA, the factors responsible for and the incidence of these two conditions should be assessed. This study analyzed the association between SCA and STEMI in patients with STEMI before receiving catheterization in Taiwan.

## Methods

### Study background and setting

This single-center retrospective cohort study investigated the risk factors for SCA in patients with STEMI and the association of the identified risk factors with clinical outcomes. This study was performed in China Medical University Hospital (CMUH) from 2016 to 2019. The CMUH is a tertiary center in Taiwan with more than 14,000 patients presenting to the emergency department (ED). Approximately 650 patients with acute coronary syndrome (ACS) present to the ED annually. Among the 650 patients with ACS who are treated annually, coronary catheterization is performed in approximately 550, whereas percutaneous coronary intervention (PCI) is performed in approximately 450 [10].

CMUH provides 24-h medical services to rescue patients with AMI. Patients with chest pain undergo an electrocardiogram (ECG) within 10 min of their presentation to the ED, and the coronary angiography team is called upon if evidence of STEMI is obtained. The door-to-balloon time for patients with STEMI was only 62 min on average between 2016 and 2019, which is much lower than the international standard of 90 min [10].

In our ED, all patients with cardiac arrest receive resuscitation in accordance with the advanced cardiac life support guidelines [11]. Factors relating to performance of extracorporeal CPR on patients include young age (< 60 years), bystander-witnessed arrest with CPR, an initially shockable rhythm, and correctable causes, such as a cardiac etiology and no ROSC within 10–20 min of CPR. Targeted temperature management is performed for patients with SCA who remain unconscious after ROSC (Glasgow Coma Scale [GCS] score of < 8) or are unable to follow orders (GCS-Motor < 6) [11]. Patients receive a coronary angiogram after ROSC if physicians are suspicious of cardiogenic causes such as indicators of myocardial infarction (sudden chest pain and collapse or witnessed collapse) or ECG-revealed ST-segment and T-wave changes.

To ensure that the study was conducted ethically, the experimental protocols were designed on the basis of the Declaration of Helsinki principles and were approved by the Research Ethics Committee of CMUH, Taiwan.

### Participants

We retrospectively collected data from AMI patients with or without SCA who presented to CMUH from January 1, 2016, to December 31, 2019. We divided the patients into two groups: the SCA group and the non-SCA group. In the initial phase, we enrolled patients with ACS. We excluded patients with NSTEMI who did not receive PCI and who did not have coronary angiography–documented CAD. Only patients with STEMI who received PCI such as stenting or balloon dilatation were included in the non-SCA group. The diagnosis of ACS was made by a cardiologist on the basis of the 2018 guidelines of the Taiwan Society of Cardiology [12]. STEMI was diagnosed using both clinical (symptoms of ischemia persisting for 20 min or longer) and ECG criteria (presumed new left bundle branch block or ST-segment elevation of 1 mm or more in the following areas: two or more anterior leads, two or more inferior leads, or two or more posterior leads).

For including patients in the SCA group, we retrospectively collected data from patients with ACS who experienced SCA and were resuscitated at our ED. Those with suspected cardiac origin of their arrest were initially included. Patients without ROSC were excluded. Those younger than 18 years, who did not undergo PCI, and who did not have coronary angiography–documented CAD were also excluded from this study.

### Data collection

Data were retrieved from the electronic medical records of CMUH. Data on resuscitation, including prehospital features (such as witness status, bystander CPR, and CPR duration), were obtained. The patients’ demographic data, including sex, age, and underlying disease (such as hypertension, diabetes, prior CAD, cerebrovascular accident, chronic kidney disease, and hyperlipidemia), were recorded. The estimated glomerular filtration rate (eGFR) obtained in the ED was considered as the baseline renal function. The cardiac catheterization results, including the number of affected coronary arteries and the involvement of the left main coronary artery, were also recorded.

### Outcome measurement

The primary outcome of our study was the occurrence of SCA in the patients with STEMI. Characteristics potentially associated with SCA were examined to identify the risk factors for SCA in the patients with STEMI. The secondary outcomes were survival to discharge and survival to discharge with favorable neurological function. A cerebral performance category (CPC) of 1 (conscious and no neurological disability) or 2 (conscious, moderate neurological disability, and can work) was defined as favorable neurological function, whereas a CPC of 3, 4, and 5 was defined as unfavorable outcomes [13].

### Statistical analysis

Statistical analyses were performed using SAS 9.4 (SAS Institute, Cary, NC, USA), and p-values of < 0.05 were considered statistically significant. The demographic data of the patients with and without SCA were analyzed. Categorical characteristics are presented as numbers and percentages, and the differences were analyzed using the chi-squared test. Continuous variables are presented as mean and standard deviation, and the differences between groups were tested using the Student’s *t*-test. Nonparametrically distributed numerical data are expressed as the median and interquartile range (IQR), and the Mann–Whitney U-test was used to analyze nonnormally distributed parameters.

Possible risk factors for SCA in the patients with AMI were analyzed using logistic regression. Multivariate analysis was performed to identify independent risk factors for SCA after AMI development, with adjustment for possible confounders. The model included the variables of age, sex, diabetes, prior CAD, hypertension, eGFR, left main CAD, and the number of affected coronary arteries. We also performed a sensitivity test to identify the risk factors for OHCA.

Variables that may be associated with the post-resuscitation prognosis, including survival to discharge and survival to discharge with favorable neurological function, were also analyzed using logistic regression. The final model for the post-resuscitation prognosis was constructed with adjustment for age, sex, CPR duration, OHCA event, prior CAD, hypertension, eGFR, left main CAD, the number of affected coronary arteries, witness status, and bystander CPR.

## Results

### Baseline characteristics

**Fig. 1:**
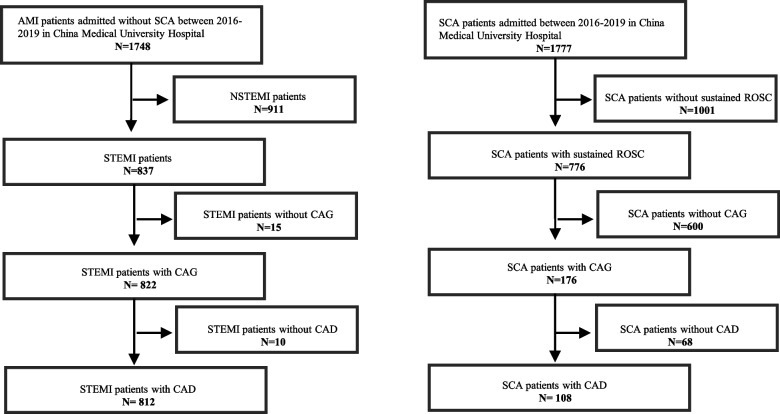
Flowchart of patient enrollment. AMI, acute myocardial infarction; SCA, sudden cardiac arrest; NSTEMI, non-ST-segment elevation myocardial infarction; STEMI, ST-segment elevation myocardial infarction; CAG, coronary angiogram; CAD, coronary heart disease; ROSC, return of spontaneous circulation

A total of 1,748 patients with AMI and 1,777 patients with SCA who presented to CMUH between January 2016 and December 2019 were included in the non-SCA and SCA groups, respectively. In the non-SCA group, we excluded 911 patients with NSTEMI, 15 patients who did not receive PCI, and 10 patients without coronary angiography–documented CAD. Finally, 812 patients with STEMI and coronary angiography–documented CAD were included in the non-SCA group. In the SCA group, 1,001 patients who could not achieve sustained ROSC were excluded. Moreover, 68 patients without coronary angiography–documented CAD and 600 patients for whom coronary angiography was not performed were excluded. Finally, 108 patients with SCA and coronary angiography–documented CAD were included in the SCA group (Fig. 1).

The average age of the patients in both groups was similar, with men being predominant in both groups. Compared with the non-SCA group, the SCA group exhibited a higher prevalence of diabetes mellitus (39.81% vs 29.93%, *p* = 0.037) and lower eGFR (51.98 ± 23.07 vs 68.37 ± 25.50, *p* < 0.001). Moreover, a higher prevalence of left main CAD was noted in the SCA group (12.96% vs 3.57%, *p* < 0.001) than in the non-SCA group. A slightly higher proportion of three-vessel disease was observed in the SCA group than in the non-SCA group; however, the difference was not statistically significant (22.22% vs 15.15%, *p* = 0.139). The comparisons of patient characteristics between the SCA and non-SCA group are provided in Table 1.

**Table 1:** Demographic characteristics of patients with acute myocardial infarction

Variables	Acute myocardial infarction		*P*-value
	SCA group (*n* = 108)	Non-SCA group (*n* = 812)	
Age, mean ± SD	61.31 ± 14.13	60.57 ± 13.39	0.589^a^
Sex, (%)			0.784^b^
M	90(83.33)	668(82.27)	
F	18(16.67)	144(17.73)	
Comorbidities			
Smoking	59(54.63)	478(58.87)	0.401^b^
Hypertension	65(60.19)	434(53.45)	0.186^b^
Diabetes mellitus	43(39.81)	243(29.93)	0.037^b^
Prior CAD	22(20.37)	121(14.90)	0.140^b^
CVA	4(3.70)	34(4.19)	1.000^b^
Chronic kidney disease	8(7.41)	46(5.67)	0.469b
Hyperlipidemia	7(6.48)	59(7.27)	0.766^b^
Creatinine, median(IQR)	1.36(1.15–1.70)	1.06(0.89–1.31)	0.019^c^
eGFR	51.98 ± 23.07	68.37 ± 25.50	< 0.001^a^
Angiographic findings			
Left main CAD	14(12.96)	29(3.57)	< 0.001^b^
Numbers of diseased vessels			0.139^b^
1	49(45.37)	427(52.59)	
2	35(32.41)	262(32.27)	
3	24(22.22)	123(15.15)	

*SCA* Sudden cardiac arrest, *SD* standard deviation, *CAD* coronary artery disease, *CVA* cerebrovascular accident, *IQR* interquartile range, *eGFR* estimated glomerular filtration rate

^a^Two-sample *t-*test

^b^Chi-squared test

^c^Mann-Whitney U-test

Of the 920 patients enrolled in this study, SCA occurred in 108 patients. The univariate analysis revealed that diabetes, lower eGFR, and left main CAD were associated with SCA. The multivariate analysis with adjustment for risk factors revealed younger age (odds ratio [OR]: 0.98; 95% confidence interval [CI], 0.96 to 0.99; *p* = 0.01), lower eGFR (OR: 0.97; 95% CI, 0.96 to 0.98; *p* < 0.001), and left main CAD (OR: 3.77; 95% CI, 1.84 to 7.72; *p* < 0.001) as significant risk factors for SCA. Similar risk factors were also noted for the patients with AMI and OHCA. Table 2 lists the results of the univariate and multivariate regression analyses.

**Table 2:** Univariate and multivariate logistic regression of cardiac arrest and out-of-hospital cardiac arrest in patients with acute myocardial infarction

Variables	Sudden cardiac arrest			
	Univariate OR (95% CI)	*P*-value	Multivariate OR (95% CI)	*P*-value
Age	1.00(0.99–1.02)	0.589	0.98(0.96–0.99)	0.010
Sex, Male	1.08(0.63–1.84)	0.784	1.25(0.69–2.26)	0.471
Diabetes mellitus	1.55(1.02–2.34)	0.038	1.16(0.72–1.89)	0.541
Prior CAD	1.46(0.88–2.42)	0.142	1.13(0.65–1.97)	0.670
Hypertension	1.32(0.87–1.98)	0.187	1.06(0.67–1.69)	0.802
eGFR	0.98(0.97–0.98)	< 0.001	0.97(0.96–0.98)	< 0.001
Left main CAD	4.02(2.05–7.88)	< 0.001	3.77(1.84–7.72)	< 0.001
No. of diseased vessels				
1 or 2	Reference	-	Reference	-
3	1.60(0.98–2.62)	0.061	1.44(0.85–2.45)	0.179
Variables	Out-of-Hospital cardiac arrest			
	Univariate OR (95% CI)	*P*-value	Multivariate OR (95% CI)	*P*-value
Age	1.01(0.99–1.03)	0.360	0.98(0.96–0.99)	0.033
Sex, Male	1.03(0.56–1.87)	0.936		
Diabetes	1.59(1.00–2.54)	0.051	1.20(0.70–2.07)	0.502
Prior CAD	1.26(0.70–2.28)	0.440	1.00(0.53–1.88)	0.988
Hypertension	1.25(0.79–1.99)	0.343	0.98(0.58–1.65)	0.923
eGFR	0.97(0.96–0.98)	< 0.001	0.97(0.96–0.98)	< 0.001
Left main CAD	3.44(1.63–7.27)	0.001	3.08(1.39–6.82)	0.005
No. of diseased vessels				
1 or 2	Reference	-	Reference	-
3	1.45(0.82–2.55)	0.199	1.27(0.69–2.34)	0.443

*OR* odds ratio, *CI* confidence interval, *CAD* coronary artery disease, *eGFR* estimated glomerular filtration rate

Among 108 patients with STEMI who experienced SCA, 57 (52.7%) patients survived to hospital discharge, and 42 (38.8%) patients had favorable neurological function at hospital discharge. Factors associated with patient prognosis after SCA are presented in Table 3. The multivariate regression analysis with adjustment revealed that the patients with advanced age (OR: 0.96; 95% CI, 0.91 to 0.99; *p* = 0.037), longer CPR duration (OR: 0.94; 95% CI, 0.91 to 0.98; *p* = 0.001), left main CAD (OR: 0.15; 95% CI, 0.03 to 0.77; *p* = 0.023), and three-vessel disease (OR: 0.25; 95% CI, 0.08 to 0.81; *p* = 0.021) had a lower survival-to-discharge rate. By contrast, the patients with witnessed cardiac arrest had a higher survival-to-discharge rate (OR: 27.38; 95% CI, 2.12 to 353.4; *p* = 0.011). Moreover, among the patients with STEMI and SCA, those with advanced age (OR: 0.90; 95% CI, 0.85 to 0.96; *p* < 0.001), longer CPR duration (OR: 0.91; 95% CI, 0.87 to 0.96; *p* < 0.001), left main CAD (OR: 0.06; 95% CI, 0.001 to 0.94; *p* = 0.044), and three-vessel disease (OR: 0.07; 95% CI, 0.01 to 0.45; *p* = 0.005) exhibited a lower probability of survival to discharge with favorable neurological function.

**Table 3:** Univariate and multivariate logistic regression of prognosis in patients with cardiac arrest after ST-segment elevation myocardial infarction

Variables	Survival discharge			
	Univariate OR (95% CI)	*P*-value	Multivariate OR (95% CI)	*P*-value
Age	0.97(0.94–0.99)	0.027	0.96(0.91–0.99)	0.037
Sex, Male	1.14(0.42–3.15)	0.795	0.72(0.20–2.63)	0.622
CPR duration(min)	0.97(0.94–0.99)	0.004	0.94(0.91–0.98)	0.001
OHCA, yes	0.57(0.24–1.40)	0.223	0.91(0.28–3.00)	0.878
CAD, yes	0.34(0.12–0.91)	0.031	0.35(0.10–1.21)	0.095
HTN, yes	0.70(0.32–1.52)	0.364	0.54(0.17–1.71)	0.291
eGFR	1.02(0.99–1.04)	0.072	1.00(0.98–1.03)	0.781
Left main CAD	0.20(0.05–0.77)	0.019	0.15(0.03–0.77)	0.023
No. of diseased vessels				
1 or 2	Reference	-	Reference	-
3	0.28(0.11–0.75)	0.011	0.25(0.08–0.81)	0.021
Witness, yes	2.99(0.55–16.12)	0.203	27.38(2.12–353.4)	0.011
Bystander, yes	0.55(0.05–6.25)	0.629	0.32(0.01–9.86)	0.513
Variables	Favorable neurological function (CPC1-2)			
	Univariate OR (95% CI)	*P*-value	Multivariate OR (95% CI)	*P*-value
Age	0.94(0.91–0.98)	0.001	0.90(0.85–0.96)	< 0.001
Sex, Male	1.40(0.48–4.06)	0.539	0.58(0.11–3.12)	0.526
CPR duration(min)	0.96(0.93–0.99)	0.005	0.91(0.87–0.96)	< 0.001
OHCA, yes	0.34(0.14–0.84)	0.019	0.27(0.06–1.19)	0.084
CAD, yes	0.37(0.13–1.10)	0.073	0.63(0.13–3.09)	0.564
HTN, yes	0.63(0.29–1.38)	0.248	1.15(0.30–4.41)	0.839
eGFR	1.03(1.01–1.06)	0.003	1.01(0.99–1.04)	0.343
Left main CAD	0.10(0.01–0.76)	0.026	0.06(0.01–0.94)	0.044
No. of diseased vessels				
1 or 2	Reference	-	Reference	-
3	0.16(0.04–0.57)	0.004	0.07(0.01–0.45)	0.005
Witness, yes	0.87(0.19–4.12)	0.865	1.97(0.18–21.90)	0.581
Bystander, yes	1.33(0.12–15.18)	0.816	4.42(0.19–102.7)	0.355

*OR* odds ratio, *CI* confidence interval, *CPR* cardiopulmonary resuscitation, *OHCA* out-of-hospital cardiac arrest, *CAD* coronary artery disease, *HTN* hypertension, *eGFR* estimated glomerular filtration rate

**Fig. 2:**
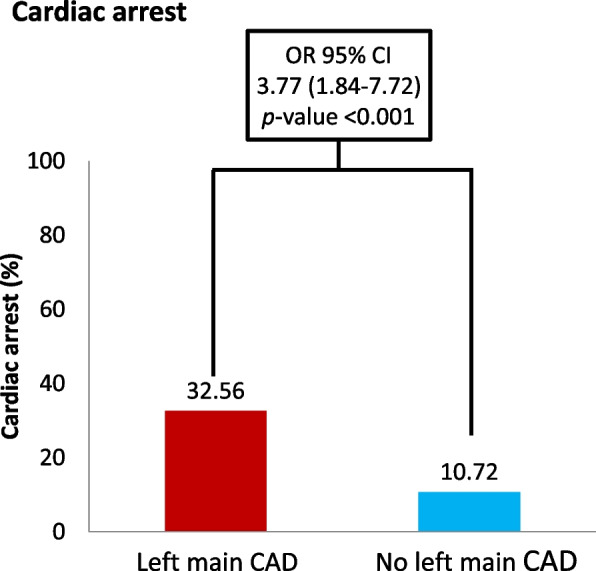
Risk of sudden cardiac arrest in patients with acute myocardial infarction with and without left main coronary artery disease. CAD, coronary artery disease

**Fig. 3:**
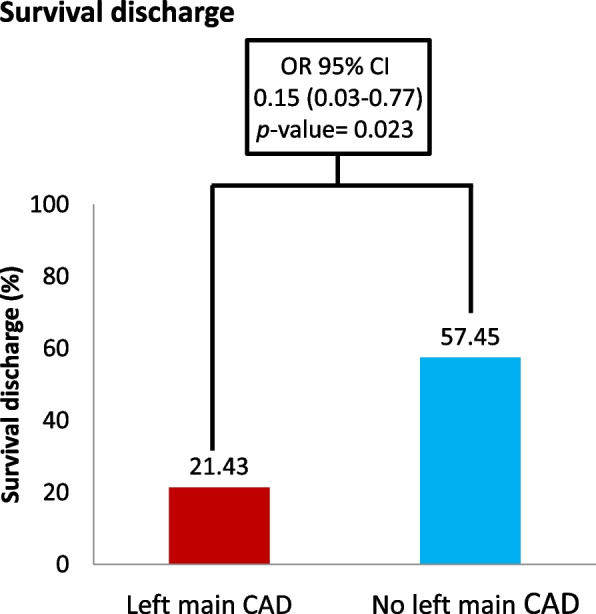
Survival-to-discharge rate after sudden cardiac arrest in patients with acute myocardial infarction with and without left main coronary artery disease. CAD, coronary artery disease

**Fig. 4:**
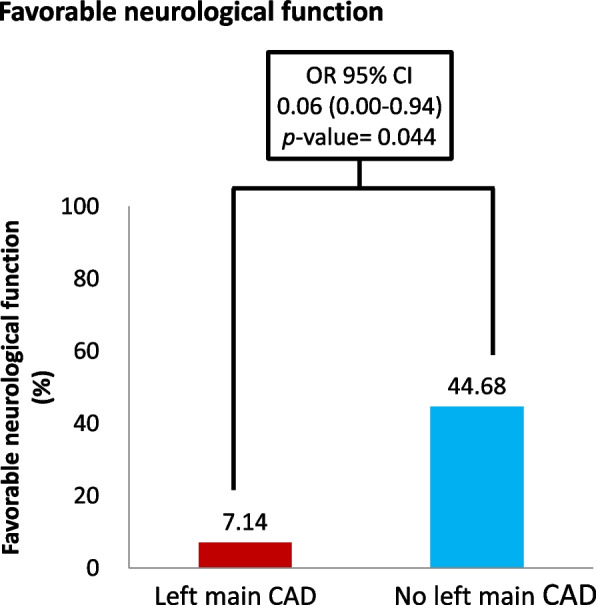
Proportion of those with survival-to-discharge who also had favorable neurological function after sudden cardiac arrest among patients with acute myocardial infarction with and without left main coronary artery disease. CAD, coronary artery disease

In the patients with STEMI, left main CAD was associated with a higher rate of SCA (32.56% vs 10.72%, *p* < 0.01, Fig. 2). Moreover, the post-resuscitation prognosis was worse in the patients with left main CAD after SCA. Left main CAD was associated with a lower survival-to-discharge rate (21.43% vs 57.45%, OR: 0.15, *p* = 0.023, Fig. 3), and only a small proportion of patients with left main CAD demonstrated favorable neurological function after SCA (7.14% vs 44.68%, OR 0.06, *p* = 0.044, Fig. 4).

## Discussion

We identified the risk factors for SCA in patients with STEMI. The patients with STEMI who experienced SCA were younger, had a lower eGFR, and were more likely to have a diagnosis of diabetes mellitus and left main CAD. Prognosis after SCA was inferior in the patients with older age and prolonged CPR duration. The patients with CAD who experienced SCA exhibited a lower survival-to-discharge rate if they had left main CAD or three-vessel disease. However, the patients with witnessed cardiac arrest had a higher survival-to-discharge rate. Furthermore, older age, prolonged CPR duration, left main CAD, and three-vessel disease were associated with unfavorable neurological function.

A study reported that OHCA patients with STEMI had worsened renal function and were more likely to have left main CAD and multivessel disease than OHCA patients without STEMI [6]. Hayiroglu et al. also reported that acute kidney injury in patients with STEMI was associated with higher in-hospital mortality and morbidity rates [14]. Cinar et al. found that creatinine is a prognostic factor for predicting the risk of in-hospital cardiac arrest for patients with STEMI-related cardiogenic shock [15]. Renal function, which is measured using creatinine levels, is also an element in the Intermountain Risk Score and has been effective in predicting short- and long-term mortality for patients with STEMI [16]. These findings indicate that worsened renal function is a major risk factor for cardiac arrest in patients with STEMI, which is consistent with our findings. Another study reported that patients with STEMI and SCA were younger, were mostly women, had severe left ventricular dysfunction, and mostly exhibited poor compliance with medications [8].In the VALIANT trial, Pouleur et al. analyzed 105 patients with SCA belonging to the autopsy group and reported that left ventricular dysfunction, heart failure, both left ventricular dysfunction and heart failure, recurrent myocardial infarction, or cardiac rupture was the major cause of death in patients who died suddenly and unexpectedly in the early period after AMI [17]. Previous studies have reported that younger patients, especially women, have less severe ischemic symptoms than older patients, resulting in a delay in seeking treatment, such as the PCI procedure. These findings explain the higher risk of sudden death in younger patients [18, 19], which was also noted in this study.

Stress may play a key role in young and middle-aged patients with STEMI. One study mentioned that combining Perceived Stress Scale and SYNTAX scores could reflect the fact that patients with both STEMI and higher stress are more likely to have an atherosclerotic coronary plaque burden and are more prone to ACS. Consequently, the chance of sudden death is also higher [20].

Zimmermann et al. reported that the survival rate and favorable neurological outcome (CPC < 2) after 1 year of the cardiac event were superior in patients with younger age, collapse–advanced cardiac life support delay < 6 min, and successful reperfusion. They also reported a higher mortality rate and CPC score in patients with asystole as the initial rhythm, reduced ejection fraction (< 30%) and creatinine level (> 1.3 mg/dL) on admission, and vasopressor usage on admission [6]. Kim et al. reported inferior neurological function in OHCA patients with left main CAD or three-vessel disease who did not receive complete revascularization [21]; similar results were also noted in this study. Our study revealed that older age, longer CPR duration, and high prevalence of left main CAD and three-vessel disease were associated with a lower survival-to-discharge rate and inferior favorable neurological function in patients with STEMI and SCA. By contrast, patients with witnessed cardiac arrest had a higher survival-to-discharge rate.

### Limitations

The strength of this study is the inclusion of a large case number with complete coronary angiography results. However, this study has several limitations. First, we enrolled only patients with SCA and coronary angiography–documented CAD. We did not perform coronary angiography for all patients with cardiac arrest, which may result in selection bias. However, we performed coronary angiography for all patients with SCA and suspicion of CAD in accordance with pre-established criteria, which may largely avoid selection bias. Second, the SYNTAX score is an additive tool according to US and European guidelines. SYNTAX score II was developed in 2013 and can predict 4-year mortality by using interactions between treatment modalities (PCI and CABG) and clinical variables [22]. Our hospital introduced the SYNTAX score system in 2021, but the scores were not included in this study because patients were enrolled from the period 2016–2019. However, the SYNTAX score system is an excellent predictive tool for patients with myocardial infarction and can be used in future studies. Third, we only evaluated creatinine level and eGFR from laboratory data. Biomarkers such as C-reactive protein were not included in this study. The C-reactive protein level in patients with STEMI has been reported to be an independent predictor of reduced left ventricular ejection fraction [23] and all-cause mortality [24] in young patients with STEMI. Further analysis including C-reactive protein can be conducted in future studies. Fourth, significantly fewer cases were included in the SCA group than in the non-SCA group, which may result in lower statistical power. However, the risk factors and prognostic factors remained statistically significant in the multivariate regression analysis with adjustment. Finally, data on several clinical factors, such as initial symptoms, symptom onset time, and long-term prognosis, were not collected in this study. Therefore, additional prospective studies are required to provide complete information.

## Conclusions

Patients with AMI who were younger, had lower eGFR, and had left main CAD were at higher risk of SCA. Older age, prolonged CPR duration, left main CAD, and three-vessel disease were associated with a lower probability of survival to discharge with favorable neurological function.

## Data Availability

The datasets generated and analyzed in this study are not publicly available due to the nondisclosure agreement of the Institutional Review Board. The datasets are available from the corresponding author upon reasonable request.

## Abbreviations

SCA: Sudden cardiac death
CHD: Coronary heart disease
AMI: Acute myocardial infarction
STEMI: ST-segment elevation myocardial infarction
NSTEMI: Non–ST-segment elevation myocardial infarction
OHCA: Out-of-hospital cardiac arrest
CPR: Cardiopulmonary resuscitation
ROSC: Return of spontaneous circulation
CAD: Coronary artery disease
CMHU: China Medical University Hospital
ED: Emergency department
ACS: Acute coronary syndrome
PCI: Percutaneous coronary intervention
ECG: Electrocardiogram
GCS: Glasgow Coma Scale
eGFR: Estimated glomerular filtration rate
CPC: Cerebral performance category
IQR: Interquartile range
OR: Odds ratio
CI: Confidence interval
